# Needs assessment study of rare diseases education for nurses and nursing students in Poland

**DOI:** 10.1186/s13023-020-01432-6

**Published:** 2020-06-29

**Authors:** Dariusz Walkowiak, Jan Domaradzki

**Affiliations:** 1 Department of Medical Law, Organization and Management in Health Care, Poznan University of Medical Sciences, Przybyszewskiego 39, 60-356 Poznań, Poland; 2 Department of Social Sciences and Humanities, Poznan University of Medical Sciences, Poznań, Poland

**Keywords:** Rare diseases, Medical education, Nurses, Students, Survey

## Abstract

**Background:**

The treatment of rare diseases in contemporary health systems appears to be steadily gaining in importance, especially as the number of their occurrence is increasing. However, the education of medical staff in their correct diagnosis and therapy seems inadequate. Our study aims to analyze the knowledge and opinions concerning rare diseases among Polish nurses and nursing students.

**Methods:**

We analyzed a group of 142 nurses and 113 nursing students, using a questionnaire comprising 28 questions about the number, examples, etiology and estimated frequency of rare diseases. Self-assessment of one’s own theoretical and practical competence in the topic, as well as the opinion of the respondents on the need for a more prominent place of rare diseases in the curricula of medical universities, were also tested. We also asked about our informants’ sources of information on rare diseases. The study was conducted between January and June 2019.

**Results:**

Although only approximately $$ \raisebox{1ex}{$1$}\!\left/ \!\raisebox{-1ex}{$3$}\right. $$rd of respondents declared having participated in university classes in rare diseases, a markedly higher number (~ 85% of nurses and ~ 75% of students) sees the need for such courses. Neither group feels well-prepared to deal with patients with rare diseases, with 75% of nurses and as many as 85% of nursing students expressing their concern in this respect. Both groups name the Internet as their major source of information.

**Conclusion:**

Both nurses and nursing students show insufficient knowledge of rare diseases, though the level of competence of the former is in many respects higher in a statistically significant way. Most respondents perceive serious deficiencies in their preparation to care for such patients. A change in university curricula seems much called for.

## Background

Intensive research into rare diseases (RDs) seems to be necessary to enable the development of new therapies for patients with one of such maladies, as well as the creation of diagnostic tests and of care procedures for the individuals affected. There is a need for intensive work related to making the public aware of the importance of treating rare diseases, since this work has been performed so far primarily by societies founded by those afflicted with a specific rare disease or by their parents, supported by scientists and doctors dealing with these diseases. Also the education of medical staff in the field of rare diseases seems to be one of the basic challenges today.

There is no single generally accepted definition of rare diseases. Richter et al. [1] identified 296 definitions from 1109 organizations, but it is hard to disagree that “RD are numerous, heterogeneous in nature, and geographically disparate” [2]. A rare disease could be defined as a wide and varied set of disorders that are characterized by each one affecting a small number of individuals in the population, being chronic and disabling, having a high morbidity and mortality rate, and for which therapeutic resources are, in general, scarce and ineffective [3]. Although individual rare diseases affect very few people, it is estimated that jointly they concern 6–8% of the world’s population [4]. This combined incidence of rare diseases means that a member of the medical personnel might never meet a patient with a specific RD, while patients with one of these diseases are encountered on a daily basis.

The problem, however, does not merely regard the health professionals’ lack of knowledge of all the diseases, which, considering the fact that their number exceeds 6000, does not seem possible or expedient [5]. What is necessary is to raise the awareness that statistically more than every twentieth patient suffers from one of the rare diseases and therefore may require a specialized, non-standard approach. But in practice patients often face challenges, due to a variety of reasons [6]. According to Black et al. [7], the “diagnostic odyssey” of rare disease patients encompasses three different periods: patient interval (starting from the first time the patient/parent notices what will later be classified as a symptom or sign of the disease); primary care interval (starting with the first patient/parent visit to primary care); and specialist care interval, the time when the diagnosis is finally made. Specialists are able to shorten each of these three periods. But, unfortunately, it is unquestionable that patients with rare diseases face diagnostic delays. This issue was for the first time presented in a large-scale survey of eight rare diseases conducted by EURORDIS in 17 European countries – Rare Diseases Europe [8] [Prader-Willi syndrome (PWS), Marfan syndrome (MFS), Crohn’s disease (CD), Duchenne muscular dystrophy (DMD), tuberous sclerosis (TS), cystic fibrosis (CF), Ehlers-Danlos syndrome (EDS) and fragile X syndrome (FRX)]. To identify the main causes of diagnostic delay, questionnaires were mailed to patients’ organizations dealing with rare diseases, with 5.980 questionnaires answered. Half of the respondents affected by FRX reported a delay of at least 2.8 years between the first appearance of symptoms and obtaining a correct diagnosis. Half of the respondents affected by EDS reported a delay of at least 14 years. Before the final diagnosis, 41% of the patients were diagnosed incorrectly and the others had no diagnosis. Incorrect diagnosis led to futile medical interventions: 16% of the patients had surgery, 33% did not receive appropriate medical treatment, and 10% were given psychological care on the assumption that symptoms were psychosomatic. Patients who initially received a false psychological or psychiatric diagnosis experienced longer delays in diagnosis. Respondents affected by TS and CF reported delays four times longer if they initially received a psychological or psychiatric misdiagnosis, and MFS patients reported delays ten times longer in the same situation. As a result, 19% of all the analyzed patients reported a loss of confidence in the healthcare system. Subsequent studies carried out by organizations of patients with rare diseases confirmed these findings [9, 10].

A recent study conducted in the USA on autosomal dominant tubulo-interstitial kidney disease (ADTKD) has shown that 25% of all parents of children diagnosed in the years 1996–2017 bypassed their children’s physicians and established direct contact with an academic center specializing in ADTKD since they would otherwise have remained without diagnosis [11]. In the case of acromegaly, a meta-analysis conducted in 2017 showed that the median duration of symptoms until diagnosis was 4.5 to 5 years (range 1–25 years) [12]. There is a consensus that a significant group of patients with Niemann–Pick disease type C remains undiagnosed or misdiagnosed [13]. In a recently published study on patients with vascular Ehlers-Danlos syndrome (vEDS), 25% of patients replied negatively to a question whether their physician explained their condition to them, or instructed them on how to manage it. When they were asked about the frustrating aspects of the vEDS diagnosis, *No cure or treatment available* was the most frequent response (64.5%), followed by the statement that *Emergency rooms do not know what vEDS is* (61.8%) [14].

From the point of view of shaping the health care system and training specialists, the key finding, after delays in the diagnosis of RD, was that even diagnosed patients and their families were not provided with enough information on all aspects of their condition, both at first diagnosis and subsequently. Patients and their families are expecting this information to be provided in a range of formats and at various levels of medical and scientific detail to ensure a full understanding and informed decision making. Parents of children with rare diseases are often frustrated by a lack of knowledge exhibited by health professionals [15]. Early diagnosis can lead to the end of the exhausting “diagnostic odyssey”, at least in the first stage. It can help inform family planning, especially in the case of inherited diseases where several children in a family may be affected before diagnosis; it informs prognosis; and finally, it assists the patient and family in gaining access to social and educational support [16]. But even diagnosed patients with rare diseases have a low chance of treatment, because in the case of over 90% of rare diseases no therapy is available [17].

At the same time, while earlier studies focused on knowledge and awareness on rare diseases among medical students and general practitioners [18–25], there is an urgent need of such training of all healthcare professionals, including nurses who are at the front line of the entire process of caring for RD patient and their families. Although the role of nurses in patient care systems varies from country to country [26–28], the relationship between the number and the level of education of nurses and patient outcomes tends to be well documented [29–32]. It seems, therefore, that the nurses’ knowledge of rare diseases may affect the treatment of patients with such diseases, probably at each of the three periods described by Black [6].

Depending on the local education system, general care nurses in the European Union member states may be trained, in vocational schools, colleges or at universities (where there exist three qualification levels: Associate degree, BA degree and MA degree) [33–35]. Poland is among 16 EU members educating nurses only at the higher education level. Among the neighbouring countries, Slovakia (BA degree and MA degree) has a similar educational system as Poland (BA degree and MA degree), while in Germany general care nurses are educated at the vocational school level, and the Czech Republic and Lithuania educate general care nurses at universities (BA degree) and vocational colleges. On the other hand, while there are many similarities in higher nursing education between different European countries [26, 34–37], some of the new EU member states do not perceive nursing as a totally independent academic discipline and it is still under the strong influence of the medical profession [38]. Meanwhile, the Polish system of postgraduate training for nurses rests on three main pillars: 1. specialization trainings, which provide nurses with specialized qualifications in a particular field of nursing and specialist’s degree; 2. qualification courses, which provide nurses with knowledge and skills in a defined nursing area; and 3. specialized courses, which provide nurses with qualifications for performing particular nursing, preventive, diagnostic, therapeutic or rehabilitative activities, i.e. endoscopy, wound healing, preventive vaccinations or caring for a patient with intestinal stoma [39]. It is significant, because during the last two decades 84% of nurses completed some form of postgraduate training, and 10.2% of all registered nurses obtained a specialist degree [34, 35].

At the same time, although Polish nursing students receive classes in clinical genetics for at least two semesters where they study about some genetic diseases (i.e. Huntington disease, CF, sickle cell disease, Niemann-Pick disease, neurofibromatosis, Pompe disease or PKU), methods and types of materials used in genetic laboratory diagnostics, they do not receive training in clinical genetics ambulatories. Nevertheless, it is in accord with an Annex to Council Directive 77/453/EEC of 27 June 1977 specifying training leading to the award of a diploma, which states that formal qualification of nurses does not provide for courses on rare diseases or genetics [40]. Moreover, each of twenty two specialization trainings for nurses supervised by the Polish Minister of Health and devised by the Center of Postgraduate Education for Nurses and Midwives in Warsaw and local chambers of nurses and midwives give very limited information on RD; while during specializations it is only a 1 h lecture dedicated to the National Rare Disease Plan and education in the field of RD, and neither qualification courses nor specialized courses give any information on caring for RD patients [36]. Moreover, there are no other specialized courses or teaching programs for nurses in RD and related subjects available in the country.

This is important because although it is mostly molecular geneticists who perform genetic testing on RD there can be observed an increasing role of nurses in diagnosing and managing of genetic disorders [41, 42]. As most RD occur shortly after birth or in early childhood and Poland currently screens for 28 rare diseases [43], Polish nurses are obliged to know the diagnostic methods of inborn disorders and they participate in prenatal and postnatal diagnostic tests. Moreover, because diagnosing RD is very difficult and many physicians do not possess knowledge on RD, the role of nurses in the diagnostic process is crucial. Because they are often the first point of contact for RD patients and their families, nurses know that listening to RD patients may be the only available tool for their diagnosis [44–46]. Moreover, the role of nurses is vital in the entire process of caring for RD patients. Not only do they participate in prenatal care and are involved in all aspects of the newborn screening process but they also help RD patients and their families in coping with the diagnosis. It is also nurses who are best suited to evaluate patients’ psychological distress resulting from the diagnosis and to monitor its psychosocial consequences. In addition, they fulfill an important role as supporters and counselors that can guide RD families through the diagnostic, therapeutic and rehabilitation process. Finally, nurses play a crucial role as health educators that can spread knowledge on RD to the entire community [46]. For all these reasons nurses should be aware of the existence of such diseases and should possess knowledge on RD [46, 47].

Thus, purpose of this article was twofold. Firstly, we aimed to assess the knowledge and awareness of rare diseases among working and future nurses. Secondly, we attempted to determine if there are differences in the knowledge of rare diseases between working nurses and nursing students, and what the main sources of information about rare diseases are. The main working hypotheses were:

1. As a result of inadequate education and formal training on RD of nursing students and nurses, their knowledge does not increase during their professional career.

2. The main source of knowledge on RD is the Internet.

3. Both nursing students and nurses are unprepared for caring for RD patients.

## Methods

The study was conducted between January and June 2019 among the students of the Poznan University of Medical Sciences, Poland, and among nurses taking their specialization courses. The student participants were recruited during regular classes. All the professionally active nurse participants were recruited during specialization courses for nurses organized by the Poznan District Chamber of Nurses and Midwives – these courses are not compulsory, and their participants are nurses who wish to progress professionally and possibly gain a promotion. A standard questionnaire was used, comprising the topics based on literature review and the objective of the study.

The process of elaborating the questionnaire followed the guidelines of the European Statistical System [48]. First, during a focus-group meeting five nursing students and five nurses with two geneticists and one sociologist elaborated a list of important issues on RD which resulted in developing a questionnaire which was assessed by three external reviewers (one nurse, one geneticists and one sociologist). Second, the questionnaire was pre-tested in face-to-face meetings with another five nursing students and five nurses. Based on the pilot study, three question were reformulated. The final version of the questionnaire was again evaluated by another three external reviewers from the same specialties.

The questionnaire included four groups of questions. The first group consisted of ten questions regarding the knowledge, definition, number and the estimated incidence of rare diseases. Other questions concerned epidemiological problems: in which age group the first symptoms of rare diseases are observed, and how many people suffer from rare diseases worldwide, in the EU and in Poland. Two questions concerned RD etiology. In addition, participants were asked to identify rare diseases in a list of twenty-eight diseases, which alongside eighteen rare diseases included also ten other common disorders. Examples of rare diseases were selected either for being commonly known (i.e. progeria, CF, Huntington’s disease or sickle cell disease), or by virtue of being included in medical university curricula (i.e. Niemann-Pick disease, neurofibromatosis, Pompe disease, phenylketonuria (PKU)). The second set of questions focused on organizational problems and comprised six items. The respondents were asked about the name of the European website containing information about rare diseases and orphan drugs, and whether Poland has a national register of patients with rare diseases. They were also asked about the percentage of rare diseases that can be treated, whether orphan drugs are reimbursed in Poland, whether rare diseases should be considered an important aspect of the Polish health policy, and which specialists should possess special knowledge of rare diseases. The third group contained six questions related to the participants’ self-assessment of knowledge and competence in the field of rare diseases. Respondents were asked if they had any RD classes, how they assessed their knowledge of rare diseases, and if they wished to expand it. The authors also wanted to know if participants felt a need to include a compulsory RD course in medical programs. The respondents were also asked about the source of their knowledge of rare diseases, and about their readiness to care for patients with such a disease. The last set, comprising six questions, concerned the respondents’ gender, year of study in the case of students or years of working experience in the case of nurses, their marital status and place of residence, but also whether the respondents had ever met a person with an RD and if they had a relative suffering from such a disease. A reliability analysis of selected rare diseases was carried out on a group of 600 students of different medical faculties. All items were significantly associated with one another and showed Cronbach’s α =0.76, 95%CI (0.735–0787) and McDonald’s ω = 0.78.

The results were presented as descriptive statistics. Differences in proportion were tested with the Likelihood Ratio Chi-square, or Fisher’s exact and Chi-square test with Yates correction test if any cells had an expected count smaller than 5. A statistical analysis of possible answers’ differences between both study groups was performed using the Mann-Whitney U test. The level of significance was set at *p* < 0.05. Statistical analysis was performed using STATISTICA v. 13.1 (TIBCO, Palo Alto, USA) and JASP 0.12.2.

## Results

Out of all the 120 students approached, 113 (94.2%) completed the questionnaire. Feedback on surveys from fourth-year students was 56/59 (94.9%) and from the fifth year – 57/61 (93.4%). The sample consisted of 104 females (92%) and 9 males (8%), all of Polish origin (Table 1). All working nurses during their specialization courses (142) completed the survey. The sample consisted of 140 females (98.6%) and 2 males (1.4%), all of Polish origin. Almost half of them (48.4%) had been working as nurses for more than 20 years.

**Table 1:** Socio-demographic characteristics of students and nurses

Characteristics	N (%)	
	Students	Nurses
**Year of study**		
4th	56 (49.6)	
5th	57 (50.4)	
**Years of professional experience**		
Less than 1		0
1–5		26 (16.8)
6–10		18 (11.6)
11–15		14 (9)
16–20		22 (14.2)
More than 20		75 (48.4)
**Gender**		
Female	104 (92)	153 (98.7)
Male	9 (8)	2 (1.3)
**Have you ever met a person suffering from RD**		
Yes	38 (33.6)	99 (63.9)
No	54 (47.8)	47 (30.3)
I do not know	21 (18.6)	9 (5.8)
**Is anyone in your family suffering from RD?**		
Yes	4 (3.5)	23 (14.8)
No	99 (87.6)	124 (80)
I do not know	10 (8.9)	8 (5.2)

More than 90% of respondents in both study groups declared having heard the term “rare disease” (Table 2). The main cause of rare diseases was known to 59.3% of students and to 67.1% of nurses. Only 3.5% of students and 8.4% of nurses correctly estimated the number of patients suffering from rare diseases in Poland. The correct number of rare diseases was indicated by 6.2% of students and 12.3% of nurses. Statistical tests showed differences in the structure of answers to individual questions in both groups (the statistical significance of differences is presented in the right column). Except for the question on the percentage of rare diseases of genetic origin, the nurses usually provided more correct answers than the student group. In the authors’ opinion, the analysis of the distribution of answers may, however, indicate to some extent either the respondents’ attempts to adapt to the expectations of the authors of the survey, or a random selection of answers (as shown e.g. in the differences in the distribution of answers to the questions about the incidence of RD in the world and in Poland).

**Table 2:** Participants’ knowledge about rare diseases

Items	Students N(%)	Nurses N(%)	*p* - value
Have you ever heard the term ‘rare diseases’?			0.35
Yes	107 (94.7)	141 (91)	
No	6 (5.3)	14 (9)	
Rare disease is one that affects less than:			0.004
1 person in 1000	17 (15)	19 (12.2)	
**1 person in 2000**	**8 (7.1)**	**18 (11.6)**	
1 person in 3000	1 (0.9)	6 (3.9)	
1 person in 5000	5 (4.4)	17 (11)	
1 person in 10,000	52 (46)	78 (50.3)	
I do not know	30 (26.6)	17 (11)	
What is the estimated number of rare diseases?			0.004
100–500	17 (15)	23 (14.8)	
1000-2000	37 (32.7)	22 (14.2)	
3000-5000	14 (12.4)	18 (11.6)	
**6000-8000**	**7 (6.2)**	**19 (12.3)**	
9000-10,000	6 (5.3)	7 (4.5)	
Over 10,000	9 (8)	29 (18.7)	
I do not know	23 (20.4)	37 (23.9)	
In what age group do rare diseases most frequently appear?			0.07
Newborns	29 (25.7)	20 (12.9)	
**Children**	**42 (37.2)**	**77 (49.7)**	
Adolescents	3 (2.6)	4 (2.6)	
Adults	6 (5.3)	14 (9)	
They are present in all age groups equally	21 (18.6)	29 (18.7)	
I do not know	12 (10.6)	11 (7.1)	
How many people suffer from rare diseases worldwide?			0.004
10–15,000,000	23 (20.3)	33 (21.3)	
50–75,000,000	35 (31)	20 (12.9)	
100–150,000,000	15 (13.3)	19 (12.3)	
200–250,000,000	5 (4.4)	9 (5.8)	
**300–350,000,000**	**7 (6.2)**	**27 (17.4)**	
Over 500,000,000	1 (0.9)	4 (2.6)	
I do not know	27 (23.9)	43 (27.7)	
How many people suffer from rare diseases in Poland?			0.78
500–1000	22 (19.5)	27 (17.4)	
10–15,000	28 (24.8)	34 (22)	
50–75,000	17 (15.1)	15 (9.7)	
100–150,000	11 (9.7)	17 (11)	
300–500,000	5 (4.4)	3 (1.9)	
1000,000	2 (1.8)	5 (3.2)	
**2–3000,000**	**4 (3.5)**	**13 (8.4)**	
Over 5000,000	0	3 (1.9)	
I do not know	24 (21.2)	38 (24.5)	
What is the most common cause of rare diseases?			0.62
Infectious and bacterial	5 (4.4)	3 (1.9)	
**Genetic**	**67 (59.3)**	**104 (67.1)**	
Autoimmune	26 (23)	35 (22.6)	
Mitochondrial	3 (2.7)	3 (1.9)	
Environmental	1 (0.9)	1 (0.7)	
I do not know	11 (9.7)	9 (5.8)	
What percentage of rare diseases are of genetic origin?			0.78
5–10%	19 (16.8)	32 (20.7)	
20%	27 (23.9)	35 (22.6)	
50%	28 (24.8)	36 (23.2)	
**80%**	**30 (26.5)**	**36 (23.2)**	
100%	0	2 (1.3)	
I do not know	9 (8)	14 (9)	

Correct answers are written in boldface

From the presented list of 28 diseases (including 18 rare ones) respondents chose those that, according to them, are rare diseases (Table 3). From the list presented, the most frequently recognized RD turned out to be Huntington disease, Pompe disease and Gaucher disease. The most frequently wrongly recognized as a RD turned out to be Munchausen syndrome. A statistical analysis of the differences in the indications of individual diseases in both groups showed in ten cases the occurrence of statistically significant differences, consistently in favor of the nurses: two non-RD diseases, fibromyalgia and Munchausen syndrome, were indicated as rare diseases by students more often than by nurses. In eight cases of rare diseases: CF, haemophilia, neurofibromatosis, Huntington disease, DMD, mucopolysaccharidoses, osteogenesis imperfecta and PKU, nurses correctly recognized them as such more often than the students.

**Table 3:** Which of the following diseases are considered to be rare in Poland?

Items	Students N(%)	Nurses N(%)	*p* - value
**Sickle cell anemia**	6 (5.3)	15 (9.7)	
**Cystic fibrosis**	15 (13.3)	43 (27.7)	0.005
**Acromegaly**	19 (16.8)	23 (14.8)	
**Haemophilia**	9 (8)	33 (21.3)	0.003
Down syndrome	2 (1.8)	5 (3.2)	
**Niemann-Pick disease**	56 (49.6)	64 (41.3)	
Halitosis	12 (10.6)	12 (7.7)	
Glaucoma	0	1 (0.7)	
**Progeria**	32 (28.3)	43 (27.7)	
**Neurofibromatosis**	10 (8.9)	33 (21.3)	0.009
**Craniodiaphyseal dysplasia**	18 (15.9)	26 (18.3)	
Cerebral palsy	6 (5.3)	3 (1.9)	
Fibromyalgia	38 (33.6)	28 (18.1)	0.004
**Huntington disease**	36 (31.9)	78 (50.3)	0.003
**Duchenne muscular dystrophy**	35 (31)	68 (43.9)	0.03
Acquired immunodeficiency syndrome	6 (5.3)	9 (5.8)	
Munchausen syndrome	59 (52.2)	62 (40)	0.05
**Mucopolysaccharidoses**	19 (16.8)	42 (27.1)	0.05
**Achondroplasia**	21 (18.6)	32 (20.7)	
Crohn’s disease	12 (10.6)	20 (12.9)	
**Pompe disease**	58 (51.3)	75 (48.4)	
**Gaucher disease**	56 (49.6)	70 (45.2)	
**Fragile X syndrome**	48 (42.5)	65 (41.9)	
**Marfan syndrome**	43 (38.1)	60 (38.7)	
Schizophrenia	1 (0.9)	1 (0.7)	
Alzheimer’s disease	1 (0.9)	1 (0.7)	
**Osteogenesis imperfecta**	18 (15.9)	62 (40)	0.0000
**Phenylketonuria**	14 (12.4)	42 (27.1)	0.004

Nurses more often than students were of the opinion that rare diseases constitute a serious public health issue (Table 4). The difference was statistically significant. A similar difference was observed when we asked for the name of the European website providing information about rare diseases and orphan drugs. Nurses more often than students (18.1 to 0.9%) know that it is Orphanet. Most nurses know that only some orphan drugs are reimbursed in Poland. The majority of both nurses and students were of the opinion that Poland has a central register of RD patients, which is not true.

**Table 4:** Participants’ knowledge about the healthcare system for RD patients

Items	Students N(%)	Nurses N(%)	*p* - value
What is the name of the European website providing information about RD and orphan drugs?			0
Rare Disease Foundation	3 (2.7)	6 (3.9)	
NORD	4 (3.5)	1 (0.7)	
EURORDIS	11 (9.7)	29 (18.7)	
R.A.R.E	5 (4.4)	5 (3.2)	
**Orphanet**	**1 (0.9)**	**28 (18.1)**	
Global Genes	1 (0.9)	3 (1.9)	
I do not know	88 (77.9)	83 (53.5)	
Is there a central register of RD patients in Poland?			0.31
Yes	64 (56.6)	100 (64.5)	
**No**	**10 (8.9)**	**8 (5.2)**	
I do not know	39 (34.5)	47 (30.3)	
What percentage of rare disease can be treated with drugs?			0.14
0%	6 (5.3)	3 (1.9)	
**5%**	**30 (26.5)**	**58 (37.4)**	
10%	22 (19.5)	23 (14.8)	
15%	16 (14.2)	19 (12.3)	
20%	11 (9.7)	11 (7.1)	
50%	1 (0.9)	7 (4.5)	
I do not know	27 (23.9)	34 (22)	
Are orphan drugs reimbursed in Poland?			0
Yes	2 (1.8)	4 (2.6)	
**Yes, some**	**50 (44.3)**	**83 (53.6)**	
No	15 (13.3)	46 (29.7)	
I do not know	46 (40.7)	22 (14.2)	
Do RD constitute a serious public health issue?			0.0001
Absolutely yes	29 (25.7)	83 (53.5)	
Yes	67 (59.3)	61 (39.4)	
No	6 (5.3)	6 (3.9)	
Definitely not	1 (0.9)	0	
I do not know	10 (8.8)	5 (3.2)	

In our study group we have not found a single person who is absolutely sure that they are prepared for caring for patients with rare diseases, just as we have not found a single nurse who rated their own knowledge about rare diseases as very good (whereas 4 students chose that option) (Table 5). However, we must admit that nurses still feel better prepared for caring for RD patients than students. More nurses than students are of the opinion that there should be a mandatory course on rare diseases in the medical curricula. Almost one-fourth of students declared that they were not searching for information about rare diseases, while only 1.3% of nurses declared this. To some extent, it is surprising, given the large age difference in both groups, that it is the nurses who treat the Internet as the primary source of information about rare diseases more often than students (79.4% vs. 54.9%) .

**Table 5:** Participants’ self-assessment of their knowledge about RD

Items	Students N(%)	Nurses N(%)	p
How would you rate your knowledge about rare diseases?			0.006
Very good	4 (3.5)	0	
Fair enough	2 (1.8)	4 (2.6)	
Insufficient	46 (40.7)	87 (56.1)	
Very poor	61 (54)	64 (41.3)	
Do you feel prepared for caring for a patient with a rare disease?			0.0000
Definitely	0	0	
Rather yes	3 (2.7)	26 (16.8)	
Rather not	51 (45.1)	72 (46.4)	
Definitely not	44 (38.9)	48 (31)	
I do not know	15 (13.3)	9 (5.8)	
Would you like to broaden your knowledge about rare diseases?			0.15
Yes	94 (83.2)	141 (91)	
No	3 (2.7)	3 (1.9)	
I do not know	16 (14.2)	11 (7.1)	
Do you think that there should be a mandatory course on rare diseases in the medical curricula?			0.0000
Definitely	26 (23)	61 (39.4)	
Rather yes	60 (53.1)	71 (45.8)	
Rather not	12 (10.6)	10 (6.4)	
Definitely not	2 (1.8)	0	
I do not know	13 (11.5)	13 (8.4)	
Did you / do you have any classes about rare disease during your studies?			0.29
Yes	36 (31.9)	57 (36.8)	
No	60 (53.1)	84 (54.2)	
I do not know	17 (15)	14 (9)	
Where do you / did you get your knowledge about rare diseases from?			0
Mandatory courses at the university	12 (10.6)	27 (17.4)	
Facultative courses at the university	9 (8)	10 (6.5)	
Scientific literature and research	15 (13.3)	34 (21.9)	
Scientific conferences, symposia	7 (6.2)	23 (14.8)	
Internet	62 (54.9)	123 (79.4)	
Other	3 (2.7)	13 (8.4)	
I do not search for such information	27 (23.9)	2 (1.3)	

## Discussion

An important challenge faced by patients with rare diseases and their families is the general lack of awareness among the members of professional medical teams about the number of existing rare diseases, their incidence, the variety of symptoms, and the usually devastating impact that an undiagnosed or late-diagnosed rare disease can have on a patient’s life. Many specialists are convinced that they will rarely encounter a rare disease in their professional careers and it is simply not possible for such a patient to get to them. In our study more than 90% of participants either had no idea of the number of patients suffering from rare diseases in our country or underestimated it, whereas 59.4% of students and 49.1% of nurses believed that the total incidence of all rare diseases was lower than 0.2%. If this were the case, a patient with an RD would be a relatively rare guest in any of the links of the healthcare system. Unfortunately, however, such patients are at least thirty times more numerous.

To the best of our knowledge, ours is the first study concerned with the knowledge and opinions about rare diseases among nurses and nursing students. Even though in 2015 Ramalle-Gómara et al. analyzed the knowledge of rare diseases among nursing students as one of the four researched groups of university students, the construction of the questionnaire was rather superficial. Still, it turned out that out of 102 nursing students, only 17% knew the definition of rare diseases, and only 42.5% were able to mention at least one rare disease [49]. Earlier studies conducted among future medical professionals have shown that there is a problem in educating students in rare diseases and the need for improvement [18–20]. The students’ knowledge appears fragmentary, incomplete and often unrelated to the year of study of the respondents [21, 22], which proves that the acquisition of this knowledge is less determined by the curricula and more by the interest and the inquisitiveness of the student. The need for changes in the medical education system results from the fact that future physicians themselves do not feel prepared to care for a patient with an RD [23]. The results of our study mostly confirm the findings of previous studies, pointing to the lack of knowledge about rare diseases among medical students, but also show the students’ recognition of the problem as important for public health, while at the same time revealing the willingness of the majority of students to broaden their knowledge. The lack of appropriate knowledge of issues related to rare diseases has unfortunately been confirmed by research among physicians who also lack an easy access to educational opportunities and to information resources regarding rare diseases [24]. Moreover, a significant disproportion in knowledge between general practitioners and specialists has been noted [24, 25]. It has been observed that general practitioners assess their academic education on rare diseases as insufficient [50] and the easiest strategy for them is to refer a patient to a university hospital [25].

Thus, although previous research focused on the awareness on RD among (future) physicians [18–25], this study confirms that also nursing students and nurses lack knowledge and awareness on RD [49]. It also shows that due to inadequate postgraduate training on RD, nurses deficiencies in their knowledge does not decrease significantly during their professional career. Moreover, as both nursing students and nurses felt unprepared for caring for RD patients, this study shows that there is a urgent need of including a RD module into university curricula and nursing postgraduate training. This is especially so as although all students who participated in the study had classes in clinical genetics for two semesters during the first year of their study, they did not receive any special training in RD. Also nurses in our study group received very limited information on RD. And because in Poland there are no specialized programs in RD for nurses, this educational gap increases.

Thus, our findings confirm that nursing curricula contains an insufficient amount of information about rare diseases. Such a suggestion was formulated earlier [20, 51], but our research is extended beyond future physicians. What is important is that is shows that there is, on the one hand, the need to expand knowledge, on the other – high acceptance of such a solution among the respondents. At the same time, the best solution does not seem to be an attempt to teach about one specific disease (unless as a model solution), or to publish random information about one specific RD on websites addressed to nurses (as it tends to be the case). It is not possible to teach anyone in detail how to diagnose and treat each of over 6000 existing rare diseases. Systemic solutions are needed during studies, as well as targeted information in the period when nurses start working in a specific place and decide to specialize in a specific field of nursing. And what we are able to do is to organize the process of education that ensures higher standards in RD teaching. An additional circumstance is the fact that Poland is among the 12 European countries that have adopted laws on nurse’s prescribing [52]. We believe that also postgraduate training of nurses must include more content related to rare diseases. Examples of programs for training nurses in this area are known and applicable [53].

The results of our research show that the basic source of information for working nurses is the Internet. The Internet is ubiquitous: it is used by patients with rare diseases, their families and healthcare system employees as well. In our study, 79.4% of nurses have mentioned the Internet as the main source of their knowledge about rare diseases, while only 21.9% of them look for specialist information in scientific literature. Thus, the key issue for those seeking information is to provide websites that offer verified and reliable information about RD. This is of special importance because frequently reliability of the web content on RD is dubious [54, 55].

Although this study brings a new insight into the state of knowledge of Polish nursing students and professional nurses about rare diseases, it also has a few limitations. First, although the response rate among nursing students was very high, we must admit that only two year groups of one medical university were tested, and therefore the results cannot in any manner refer to all nursing students. Second, this study represents only the opinions of those nurses who wish to develop professionally, as indicated by their decision to attend specialization courses, and who agreed to participate in the study. Consequently, the results may not reflect the opinions of other nurses and cannot be generalized. However, some advantages of this study should also be acknowledged. Most importantly, this is the first study focused on knowledge and awareness of RD among nursing students and nurses in Poland. Considering the key role of nurses in the entire process of caring for RD patients and their families, this study may stimulate further research on the topic. Moreover, it may also provoke discussion on the need of better education of all healthcare practitioners, including nurses.

All in all, we suggest that in order to overcome the educational gap identified, the following guidelines should be implemented:

1. All medical curricula should include an RD module. Additionally, using examples from other European countries, such as France, Spain or the UK, Poland should implement teaching programs in RD aiming at increasing knowledge and awareness of all healthcare professionals’ on RD.
2. All medical students and healthcare providers should be taught and trained in the basic genetics and newborn screening, including genetic laboratory diagnostics.
3. As the Internet is the main source of information on RD, e-learning programs or courses should be organized.
4. Polish web pages with reliable information on RD for healthcare professionals should be available.

These recommendations are of special importance because while some European countries, i.e. France and Spain, have fully implemented national plans/strategies for RD **as recommended by the Council of the European Union in 2009, and** others have created such plans within the timelines defined by the European Commission (i.e. Belgium, Bulgaria, Cyprus, Czech Republic, Denmark, Germany, Greece, Hungary, Ireland, Italy, Latvia, Lithuania, Portugal, Romania, Slovak Republic, Slovenia, the Netherlands, and United Kingdom), in yet other countries, including Poland, the implementation of national strategies is still in progress [43, 56, 57].

## Conclusions

The knowledge of rare diseases among nurses and nursing students seems to be insufficient. Their knowledge of both the diseases themselves and the systemic solutions for their treatment must improve rapidly. What is alarming is that while both groups declare a low training level in rare diseases and do not feel adequately prepared for caring for RD patients, less than one third of nursing students, and slightly more practicing nurses, have received any training in rare diseases during their university education or postgraduate years. Our results indicate that one way to improve this is to change the medical university curricula, but also to find methods to reach those interested in providing for them the most reliable websites and rare disease databases, as most of them search the Internet to find answers. Although nurses’ knowledge about rare diseases is greater than that of the nursing students still it is not sufficient. At the same time, a high awareness among nurses that rare diseases constitute a serious public health issue might be a good prognostic for effective changes in this respect. Thus, as the vast majority of respondents from both groups expressed their willingness to broaden their knowledge about rare diseases, it seems crucial to provide both students and nurses with university and postgraduate courses on rare diseases and to enable them to cooperate in interdisciplinary healthcare units that would support the process of decision-making and improve the quality of nursing care for RD patients and their families. Moreover, there is an urgent need to prepare practice guidelines for nurses that would help them in making informed decisions and in organizing health care for RD patients. The reason for this is that it is nurses who play an important role of health educators, helping RD patients and their families as guides and leaders of the family health teams. This is of special importance because while national strategies for rare diseases have been implemented in most European countries, and the European Commission had recommended that Poland should have such a strategy by 2013, the country still lacks it. Nevertheless, it should be acknowledged that one of the four priorities of the most recent versions of the Polish National Plan for Rare Disease created in 2017 under the leadership of the Polish Ministry of Health, is to provide access for all healthcare professional, including physicians, physiotherapists, nurses, midwives psychologists, dieticians and speech therapists, to specialist courses, postgraduate internships and specialization trainings on RD [58]. The aforementioned pillars are defined as **R**ecognition and therapy, **A**cceptance and support, **R**ehabilitation, and **E**ducation, Research, Information and Social Awareness, and the plan is said to be adopted by June 2020.

## Data Availability

The datasets used and/or analyzed in the current study are available from the corresponding author on reasonable request.

## Abbreviations

RD: Rare disease
PWS: Prader-Willi syndrome
MFS: Marfan syndrome
CD: Crohn’s disease
DMD: Duchenne muscular dystrophy
TS: Tuberous sclerosis
CF: Cystic fibrosis
EDS: Ehlers-Danlos syndrome
FRX: Fragile X syndrome
ADTDK: Autosomal dominant tubulo-interstitial kidney disease
vEDS: Ehlers-Danlos syndrome
PKU: Phenylketonuria
